# Chloride Ion Adsorption Capacity of Anion Exchange Resin in Cement Mortar

**DOI:** 10.3390/ma11040560

**Published:** 2018-04-05

**Authors:** Yunsu Lee, Hanseung Lee, Dohyun Jung, Zhengxin Chen, Seungmin Lim

**Affiliations:** Department of Architectural Engineering, Hanyang University, Ansan 15588, Korea; 23yslee@gmail.com (Y.L.); ercleehs@hanyang.ac.kr (H.L.); cirel31@gmail.com (D.J.); czxgfyx@foxmail.com (Z.C.)

**Keywords:** anion exchange resin, cement mortar, chloride adsorption

## Abstract

This paper presents the effect of anion exchange resin (AER) on the adsorption of chloride ions in cement mortar. The kinetic and equilibrium behaviors of AER were investigated in distilled water and Ca(OH)_2_ saturated solutions, and then the adsorption of chloride ions by the AER in the mortar specimen was determined. The AER was used as a partial replacement for sand in the mortar specimen. The mortar specimen was coated with epoxy, except for an exposed surface, and then immersed in a NaCl solution for 140 days. The chloride content in the mortar specimen was characterized by energy dispersive X-ray fluorescence analysis and electron probe microanalysis. The results showed that the AER could adsorb the chloride ions from the solution rapidly but had a relatively low performance when the pH of its surrounding environment increased. When the AER was mixed in the cement mortar, its chloride content was higher than that of the cement matrix around it, which confirms the chloride ion adsorption capacity of the AER.

## 1. Introduction

There are concerns with respect to the durability of reinforced concrete structures in marine environments. In marine environments with high chloride contents, chloride ions actively penetrate into the reinforced concrete, which induces corrosion in the reinforcing bars inside the concrete. Therefore, many discussions and studies have been conducted to prevent the penetration of chloride ions into concrete and inhibit the corrosion of reinforcing bars [1].

The diffusion of chloride ions into concrete is largely dependent on permeability, which is governed by porosity and pore size distribution [2,3,4]. Furthermore, hydration products in concrete can physically or chemically adsorb chloride ions, which is called the chloride binding capacity of concrete [5,6,7,8]. Therefore, it is important to consider the development of hydration products to improve chloride ingress resistance in concrete. The physical adsorption of diffused chloride ions is mainly affected by calcium silicate hydrate (C-S-H), whereas the chemical adsorption, which induces more stable compounds than those of physical adsorption, is mainly affected by the alumina ferric oxide monosulfate (AFm) phases [5,7,8]. It is known that the chlorides produced by the reaction between hydration products and chloride ions are likely bound on the AFm phases rather than on the C-S-H. Chloride binding capacity is significantly attributed to the AFm phases [8].

The AFm phases belong to a family of layered double hydroxide compounds in which anions are combined between two positively charged layers [9]. Generally, OH^−^ and SO_4_^2−^ and/or CO_3_^2−^ are combined between two layers of the AFm phases, which are formed by the hydration of Portland cement. In environments with a high chloride ion concentration, the chloride ions can be exchanged with the internal anions and combined between the layers [10,11]. Therefore, they are widely investigated to increase the amount of layered double hydroxides for improving the chloride binding capacity of concrete [12,13,14,15].

This study was focused on the applicability of anion exchange resin (AER) to adsorb chloride ions. Ion exchange resin is widely used for the removal of harmful ions in the water treatment field and in the production of ultrapure water for semiconductor and liquid crystal display (LCD) cleaning [16,17]. In the field of construction materials, the solidification treatment of ion exchange resins for the recovery of radioactive materials and heavy metals has been mostly studied [18,19,20]. Only a few studies have been conducted on the effects of ion exchange resin on cement-based composite materials. The effects of Na^+^ cation exchange resin on the hydration of C_3_S were reported in [21]. The AER-mixed mortar was found to remove chloride ions in chloride-contaminated concrete [22]. Moreover, The AER-mixed grout was used to remove chloride ions in grouted tendons [23]. However, there is still a lack of studies on the adsorption of chloride ions by the AER in cement-based composite materials. This study demonstrates the role of the AER in cement mortar and its potential to improve the durability of cementitious materials. Although the effect of the AER on the hydration and mechanical properties of cement should be evaluated for use in practice, this study focused on the behavior of chloride ions in the diffusion process. The effect of the AER on the chloride binding capacity of cement mortar was investigated through an immersion test in a NaCl solution.

## 2. Materials and Methods

### 2.1. Anion Exchange Resin

**Figure 1:**
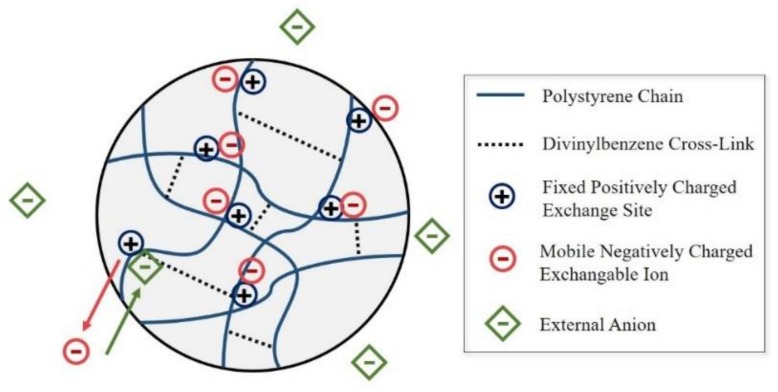
Structure of the anion exchange resin.

The structure of ion exchange resin typically has a functional group that is combined with a polymer. Depending on the charge of the functional group, it is necessary to determine whether a cation or an anion is combined with the ion exchange resin. As shown in Figure 1, the AER has a positively-charged functional group, which induces the exchange of anions depending on ion selectivity. Table 1 shows the physiochemical properties of the AER. The AER used in this study was a porous spherical bead-type resin (TRILITE SAR20) produced by Samyang. TRILITE SAR20 is composed of a N^+^(CH_3_)_2_C_2_H_4_OH functional group and a polystyrene-divinylbenzene polymer. The OH^−^ is bound to the functional group. The density of the AER is approximately 1.13, and the diameter is 300–1000 μm. The total ion exchange capacity of the AER is 1.3 eq·L^−1^.

### 2.2. Measurement of Kinetic and Equilibrium Behaviors of AER in Chloride Solutions

The direct evaluation of ion exchange reaction of the AER is challenging when it is mixed with cement mortar. If the evaluation of the reaction behavior and the equilibrium relation between the AER and chloride ions in solution is prioritized, the principle of the AER reaction in cement mortar can be inferred from the solution test. In this study, the ion exchange reaction of the AER was performed in chloride solutions. The reaction of the AER in the chloride solutions was evaluated using kinetic equations and isotherm adsorption equations. All solutions were prepared at a water-to-solid ratio of 40. The solution was stirred to a homogenous condition using a magnetic stirrer. After each reaction under the experimental conditions, the solution and ion exchange resin were separated using a vacuum filtration apparatus. The chloride concentration of the reacted solution was measured with a titrator (Metrohm Titrator 808, Metrohm, Herisau, Switzerland) using the AgNO_3_ potentiometric titration method.

Table 2 presents the experimental conditions of the kinetic test in chloride solutions containing the AER. The initial chloride ion concentration of the solution for the kinetic test was 2000 mg/L, and the kinetic property of the AER was evaluated by measuring the chloride concentration in the reacted solution over time. The chloride solutions were prepared with distilled water and a Ca(OH)_2_ saturated solution. The Ca(OH)_2_ saturated solution represented the environment of concrete pore solution. The pH of Ca(OH)_2_ saturated solution was measured using a pH meter (DKK-TOA HM-41X, DKK-TOA, Tokyo, Japan). The initial pH of Ca(OH)_2_ saturated solution was 12.34. 

The chloride concentrations of the solution were measured after the AER reacted with the chloride solution for 1, 3, 10, 30, 60, and 120 min, respectively. The chloride concentration adsorbed on the AER was calculated by subtracting the measured chloride concentration in the reacted solution from the initial chloride ion concentration. The concentration of adsorbed chloride ion over time was curve-fitted using a pseudo first-order reaction equation (Equations (1) and (2)) and a pseudo second-order reaction equation (Equations (3) and (4)) to evaluate the kinetic property of the AER [16,24,25,26,27]. While Equations (1) and (3) are the non-linearized form, Equations (2) and (4) are the linearized form.

Equations (1) and (2) are given as follows:

(1) $$Q_{t}=Q_{e}-Q_{e}{}^{-K_{1}t}$$

(2) $$\ln\left(Q_{e}-Q_{t}\right)=\ln Q_{e}-K_{1}t$$

where $Q_{t}$ is the amount of the adsorbate at time *t* (mg Cl^−^/g resin), $Q_{e}$ is the amount of adsorbate at equilibrium (mg Cl^−^/g resin), $K_{1}$ is the kinetic constant of the pseudo first-order reaction ($\min^{-1}$), and *t* is the reaction time (min).

Equations (3) and (4) are given as follows:

(3) $$Q_{t}=\frac{K_{2}Q_{e}^{2}t}{1+K_{2}Q_{e}t}$$

(4) $$\frac{t}{Q_{t}}=\frac{1}{K_{2}Q_{e}^{2}}+\frac{t}{Q_{e}}$$

where $Q_{t}$ is the amount of the adsorbate at time *t* (mg Cl^−^/g resin), $Q_{e}$ is the amount of the adsorbate at equilibrium (mg Cl^−^/g resin), $K_{2}$ is the kinetic constant of the pseudo second-order reaction (g resin/mg Cl^−^ min) and *t* is the reaction time (min).

Table 3 presents the experimental conditions of the equilibrium test in chloride solutions containing the AER. The equilibrium tests were conducted with the initial chloride ion concentration in the solution at 100, 250, 500, 1000, 2000, 5000, and 15,000 mg/L, respectively. Both distilled water and Ca(OH)_2_ saturated solution were used again as solvents. The reaction time between the AER and the chloride solution for the equilibrium test was 120 min. The reaction time was considered on the basis of the results of the kinetic test. Two hours was considered to be sufficient time to reach the equilibrium state in the distilled water and Ca(OH)_2_ saturated solution. The adsorbed chloride ions were curve-fitted depending on the free chloride ions using the Langmuir adsorption isotherm equation (Equations (5) and (6)) and the Freundlich adsorption isotherm equation (Equations (7) and (8)) to evaluate the equilibrium property of the AER [16,26,27,28]. While the Equations (5) and (7) are the non-linearized form, the Equations (6) and (8) are the linearized form.

Equations (5) and (6) are given as follows:

(5) $$Q_{e}=\frac{Q_{max}K_{L}C_{e}}{1+K_{L}C_{e}}$$

(6) $$\frac{C_{e}}{Q_{e}}=\frac{1}{Q_{max}K_{L}}+\frac{C_{e}}{Q_{max}}$$

where $Q_{e}$ is the amount of adsorbate on the adsorbent at equilibrium (mg Cl^−^/g resin), $Q_{max}$ is the maximum amount of adsorbate on the adsorbent (mg Cl^−^/g resin), $K_{L}$ is the constant of the Langmuir adsorption isotherm equation (L/mg), and $C_{e}$ is the concentration of adsorbate in the solution at equilibrium (mg/L).

Equation (4) is given as follows:

(7) $$Q_{e}=K_{F}C_{e}^{\frac{1}{n}}$$

(8) $$\ln Q_{e}=\ln K_{F}+\frac{1}{n}\ln C_{e}$$

where $Q_{e}$ is the amount of adsorbate on the adsorbent at equilibrium (mg Cl^−^/g resin), $K_{F}$ is the constant of the Freundlich adsorption isotherm equation ((mg/g resin)/(mg/L)*^n^*), $C_{e}$ is the concentration of adsorbate in the solution at equilibrium (mg/L), *n* is the experimental parameter which represent the heterogeneity of the adsorption sites.

### 2.3. Preparation of Cement Mortar Specimens

Type 1 Portland cement (Sungshin, Seoul, South Korea), Korea standard (KS) sand, and the AER were used to prepare mortar specimens according to the ASTM C 1329 standard. Table 4 presents the oxide composition of the type 1 Portland cement. Table 5 presents the mix proportions of the cement mortar. The AER was substituted by a vol % of a fine aggregate (sand), with the assumption that the density of the sand was twice that of the AER (actually, the density of the sand was approximately 2.3 times higher than that of the AER). The mortar was placed in prismatic molds of size 40 × 40 × 160 mm^3^. The mortar specimens were demolded after one day and then cured for another 27 days in an environmental chamber with constant temperature and humidity, 20 °C and 60% RH, respectively.

### 2.4. Preparation of Chloride Solutions and Samples for Immersion Test

**Figure 2:**
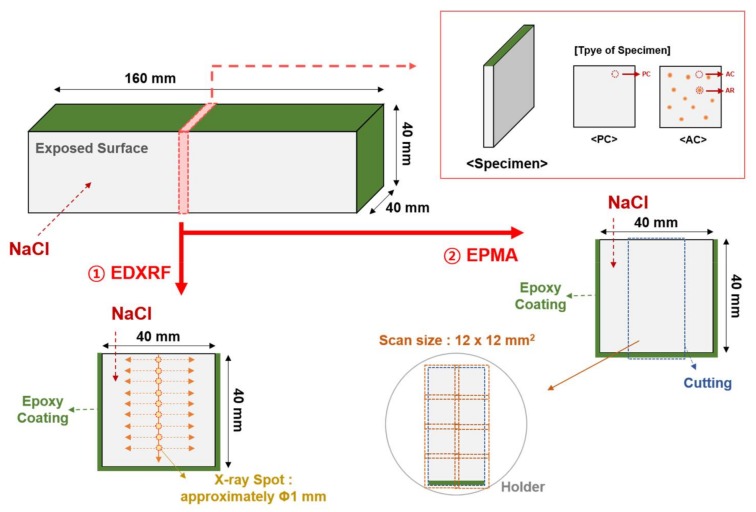
Outline of the sample preparation for X-ray analysis: EDXRF and EPMA.

A NaCl solution was prepared for an immersion test by mixing 3.2 wt % CP-grade NaCl (Daejeong, Siheung, Korea) in distilled water. The mortar specimen was coated with epoxy after 28 days of curing, except for the surface where the NaCl solution penetrates into, as shown in Figure 2. The coated mortar specimen was then immersed in the NaCl solution for 140 days. After being immersed for 140 days, the mortar specimens were cut into small plates with a thickness of approximately 5 mm using a diamond saw with a water-soluble oil. Mortar sections were subsequently immersed in isopropyl alcohol to remove the water-soluble oil for several minutes. They were polished using #1000 and #2000 SiC papers. Isopropyl alcohol was used as a lubricant to reduce the damages to the mortar sections during the polishing process. The polished specimens were stored in a chamber with 11% RH. The drying method at 100 °C was not used to avoid damage to the ion exchange resin. The chloride profiles of the polished mortar specimens were obtained using energy dispersive X-ray fluorescence (EDXRF) and electron probe microanalysis (EPMA), as shown in Figure 2.

### 2.5. Energy Dispersive X-ray Fluorescence Analysis

EDXRF analysis was conducted to investigate the major atoms in the mortar specimens according to the depth from the exposed surface using a Thermo Scientific ARL QUANT’X EDXRF spectrometer (Thermo fisher scientific, Waltham, MA, USA). The oxide contents of Na, Al, Si, S, Cl, K, and Ca were measured in a vacuum condition. The excitation of low Za (Na, Al, and Si) was performed at 4 kV, and that of low Zb (S, Cl, K, and Ca) was performed at 8 kV. The Za and Zb are the nuclear charge numbers of the cation and anion, respectively. The diameter of the X-ray spot was approximately 1 mm. The measured spots were 2, 6, 10, 14, 18, 22, 26, 30, and 34 mm from the exposed surface, as shown in Figure 2. Measurements were performed from side to side to reduce the influence of sand because chloride ingress generally occurs through the pore in the paste matrix and interfacial transition zone, not in the sand [29].

### 2.6. Electron Probe Microanalysis

EPMA was conducted to supplement the EDXRF results and obtain an image of the chloride profile. Cl and K were only analyzed to reduce the measurement time. The EPMA instrument was a Shimadzu EPMA-1720 (Shimadzu, Kyoto, Japan). The mortar specimen was cut to a size of 20 × 40 mm, as shown in Figure 2. The measured conditions were as follows: an accelerating voltage of 15 kV, current of 100 nA, pitch of 50 μm, and a unit measurement time of 40 ms point^−1^. An area of 12 × 12 mm was scanned, and then the scanned image was electronically stitched together to create a composite image [30].

## 3. Results and Discussion

### 3.1. Kinetic and Equilibrium Behaviors of the AER in Chloride Solutions

**Figure 3:**
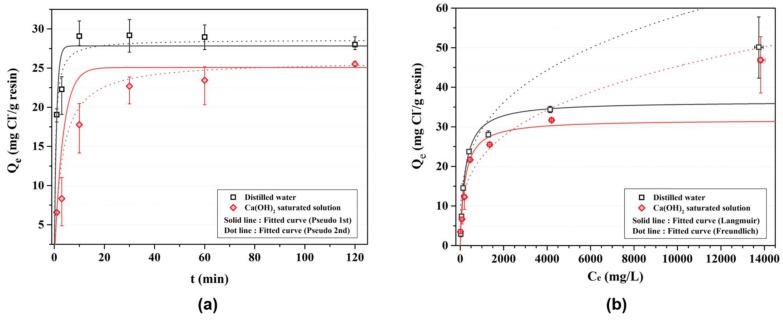
Results of the kinetic and equilibrium test of the AER in the chloride solutions—non-linear curve: (**a**) kinetic behavior of the AER; and (**b**) equilibrium behavior of the AER.

**Figure 4:**
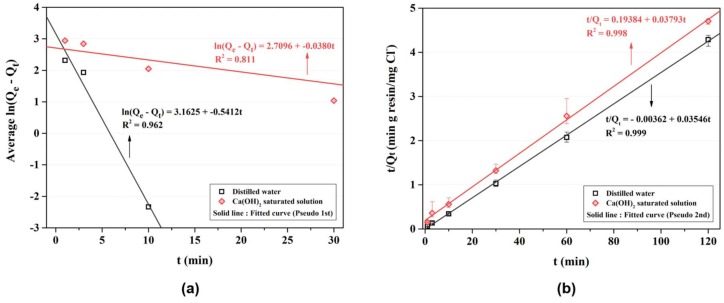
Results of the kinetic test—linear curve: (**a**) pseudo first-order reaction equation; (**b**) pseudo second-order reaction equation.

Figure 3a represents the kinetic behavior of the AER using non-linear equation (Equations (1) and (3)). Table 6 shows the experimental results of the kinetic test. Table 7 shows the kinetic parameters of the AER evaluated using the non-linear pseudo first-order and non-linear pseudo second-order reaction equations (Equations (1) and (3)), respectively. As shown in the figure, the ion exchange reaction of the AER reached the equilibrium state after approximately 10 min in the distilled water and approximately 30 to 60 min in the Ca(OH)_2_ saturated solution. After the equilibrium was reached, the chloride contents adsorbed on the AER were lower in the Ca(OH)_2_ saturated condition than in the distilled water condition. The kinetic property of the AER can be easily seen in the Figure 4. 

The pseudo first-order reaction could be observed in the polarization-dependent physisorption, whereas the pseudo second-order reaction could be observed in the chemisorption, in which strong chemical bonding occurs [10,22]. Thus, the kinetic behavior of the AER exhibited both physisorption and chemisorption in the distilled water; however, chemisorption was more dominant than physisorption in the Ca(OH)_2_ saturated solution. In the physisorption, the binding energy was dependent on the polarizability and, therefore, a weak Van der Waals interaction appeared. On the other hand, electron transfer or electron sharing occurred between the ions to form a strong bond in the chemisorption [22]. The ion exchange reaction of the AER could be regarded as chemisorption because the positively charged organic molecule N^+^(CH_3_)_2_C_2_H_4_OH functional group and anions were bonded by an electrostatic force; however, as the polarity of the organic molecule was not as strong as that of the inorganic molecule, the reversible and fast reaction characteristic of physisorption appeared to exist. Moreover, OH^−^ acted as a competing ion of Cl^−^ in the Ca(OH)_2_ saturated solution, and, thus, the time to reach the equilibrium state was delayed and the adsorbed Cl^−^ content could decrease. The linear equation was investigated further.

Figure 4 represents the kinetic behavior of the AER which is evaluated using the linear equation (Equations (2) and (4)). In the Figure 4a, the kinetic constant *K*_1_ is clearly distinguished between the types of the solution. The *K*_1_ in the distilled water and the Ca(OH)_2_ saturated solution is 0.5412 and 0.0380, respectively, which shows the kinetic performance is more predominant in the distilled water than the Ca(OH)_2_ saturated solution. When the ion adsorption is interfered with the pore diffusion, it may not be linear curve in the pseudo first reaction [31]. Since the OH^−^ can interfere with the Cl^−^ adsorption in the Ca(OH)_2_ saturated solution, the linear curve may have little agreement with pseudo first reaction. The coefficient of determination (*R*^2^) of the curve fitted by Equation (1) was similar to that of the curve fitted by Equation (3) in the distilled water; however, it was lower in the Ca(OH)_2_ saturated solution. Figure 4b represents the linear equation of pseudo second reaction. Since the reaction is generally affected by the amount of chloride ion at equilibrium and the amount of the AER, the number of active site can be identified over the time. Thus, the kinetic behavior influenced by OH^−^ decreases in the Ca(OH)_2_ saturated solution unlike the Figure 4a. The Figure 4b clearly shows the linear curve and thus it may useful to evaluate the kinetic property in the concrete environment rather than the pseudo first-order reaction.

Therefore, if there was a competing ion like in the Ca(OH)_2_ saturated condition, the ion exchange reaction of the AER could be more dependent on the chemisorption. In this study, the ion exchange reaction of the AER is as follows:

AER-OH^−^ + Cl^−^ ↔ AER-Cl^−^ + OH^−^.

Figure 3b shows the equilibrium behavior of the AER using the non-linear equation (Equations (5) and (7)). Table 8 shows the experimental results of the equilibrium test. Table 9 presents the equilibrium parameters of the AER evaluated using non-linear equations (Equations (5) and (7)). The behavior of the Langmuir adsorption isotherm is consistent with homogeneous sorption mainly due to chemisorption rather than to the Freundlich adsorption isotherm, which corresponds to the physisorption on the heterogenous surface. This is because the ion exchange reaction of the AER occurs owing to electrostatic forces when the functional groups of the AER and anions are combined [16,28].

The amount of adsorbed chloride ions was lower in the Ca(OH)_2_ saturated solution than in the distilled water across the most range of chloride concentration. The *R*^2^ of the curve fitted by non-linear Langmuir isotherm (Equation (5)) was higher than that of the curve fitted by non-linear Freundlich isotherm (Equation (7)). Considering the only *R*^2^ of the fitted curve, we can conclude that the ion exchange reaction of the AER to be more consistent with the Langmuir adsorption isotherm than with the Freundlich adsorption isotherm. However, the fitted curve (Langmuir-) appeared to underestimate at the highest chloride condition. It is showed that the fitted curve (Langmuir-) may be proper at the low chloride concentration (below 4000 mg/L) whereas the fitted curve (Freundlich-) may be suitable at the high chloride concentration (above 4000 mg/L). The linear equation was investigated further.

**Figure 5:**
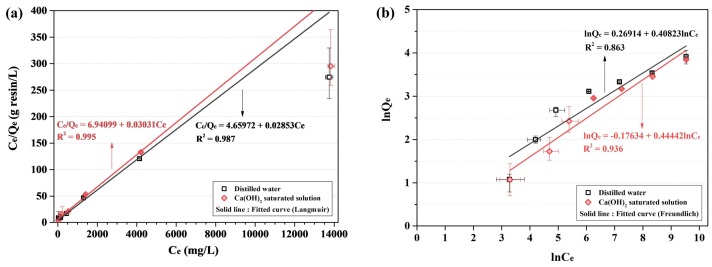

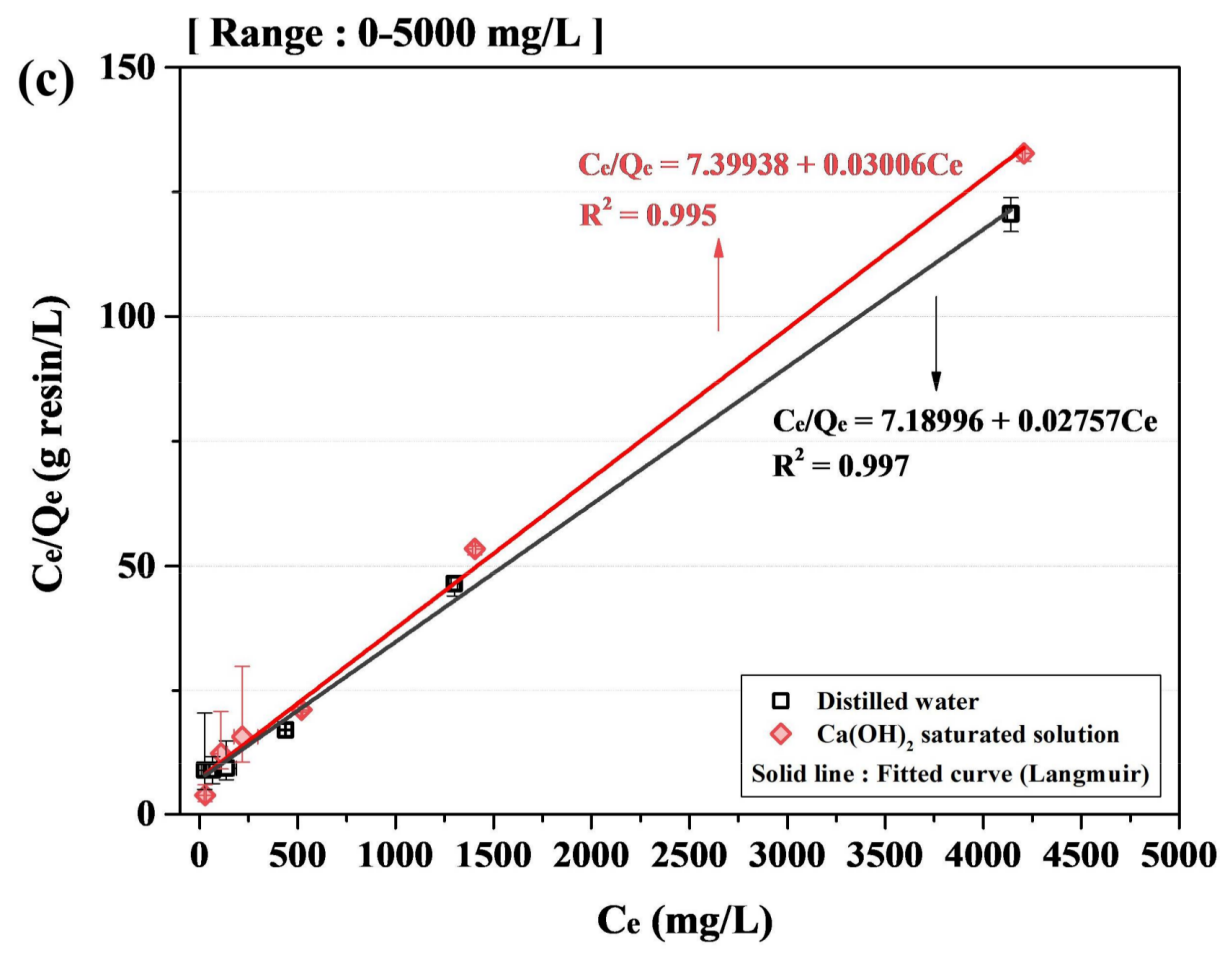
Results of the equilibrium test—linear curve: (**a**) Langmuir isotherm equation; (**b**) Freundlich isotherm equation; and (**c**) Langmuir isotherm equation (curve fitting range: 0 to 5000 mg/L).

Figure 5 represents the equilibrium behavior of the AER which is evaluated using the linear equation (Equations (6) and (8)). Figure 5a,c shows the fitted curve evaluated by linear Langmuir isotherm at the different range of chloride concentration. Figure 5b shows the fitted curve evaluated by linear Freundlich isotherm. The fitted curve in the range of 0 to 5000 mg/L was more relevant to the experimental data than that in the range of 0 to 14,000 mg/L. All the fitted curves evaluated by the linear Langmuir isotherm accorded with the data more than the fitted curve evaluated by the linear Freundlich isotherm. It is judged that the adsorption behavior of the AER fundamentally follows the homogenous adsorption of chloride on the surface. The OH^−^ ion may affect the homogenous adsorption. 

Figure 5b shows the effect of OH^−^ ion on the Freundlich isotherm. The *R*^2^ of the fitted curve in the Ca(OH)_2_ saturated solution was relatively higher than that of the fitted curve in distilled water. Since the OH^−^ ion may give rise to the heterogeneity on the surface of the AER, the Freundlich isotherm may show the good agreement in the Ca(OH)_2_ saturated solution. However, the amount of adsorbed chloride ion was low in all ranges of Ca(OH)_2_ saturated solution than distilled water although Freundlich isotherm shows the increase in the amount of adsorbate as the concentration at equilibrium increases. It is why the OH^−^ ion acts as a competing ion which is affected by the ion selectivity coefficient [32].

In conclusion, when the AER is used in the cement mortar, the adsorption of chloride ions by the AER is expected to occur more rapidly because the time to reach the adsorption equilibrium in the reaction between the AER and the chloride ions is faster than in the reaction between hydration products of cement and chloride ions [5,12]. However, the high level of pH in pore solution of the mortar specimen may reduce the chloride adsorption of the AER.

### 3.2. Energy Dispersive X-ray Fluorescence Analysis

**Figure 6:**
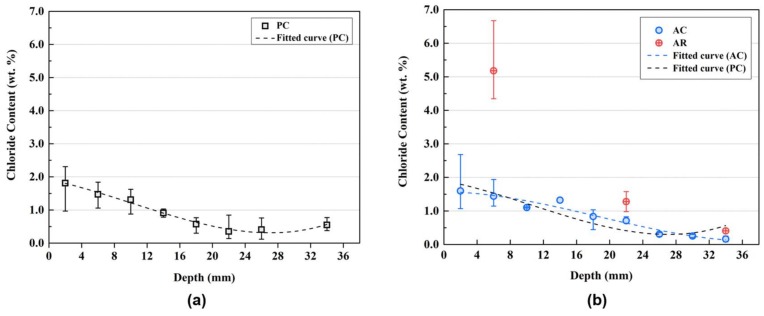
Chloride profile of the mortar specimens: (**a**) chloride content of PC; and (**b**) chloride content of AC.

**Figure 7:**
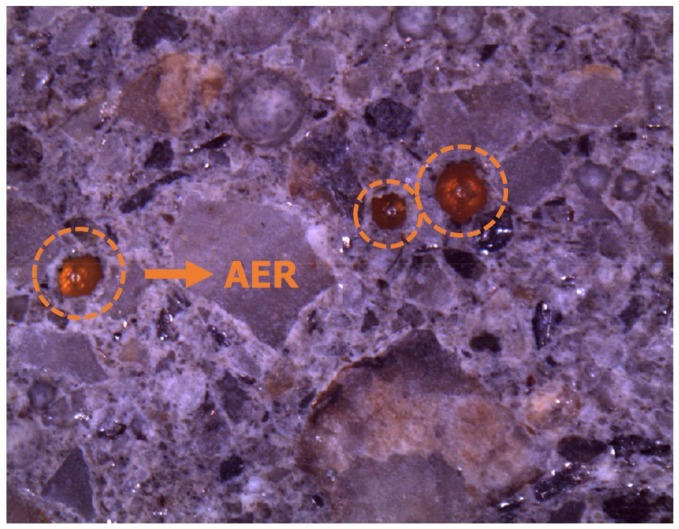
Section of the mortar specimen containing the AER.

Figure 6 shows the chloride content of the mortar specimens according to the depth from the exposed surface. PC represents the cement matrix of a plain mortar specimen without the AER. AC is the cement matrix of the mortar sample with the AER, but the location is away from the AER. AR represents the location where the AER is embedded in AC as shown in Figure 2. The section of AC is shown in Figure 7. The measured chloride content was fitted using a nonlinear curve. The fitted curve of PC was compared to that of AC to determine the effects of the AER on the changes in the chloride ion content in the paste matrix. 

**Figure 8:**
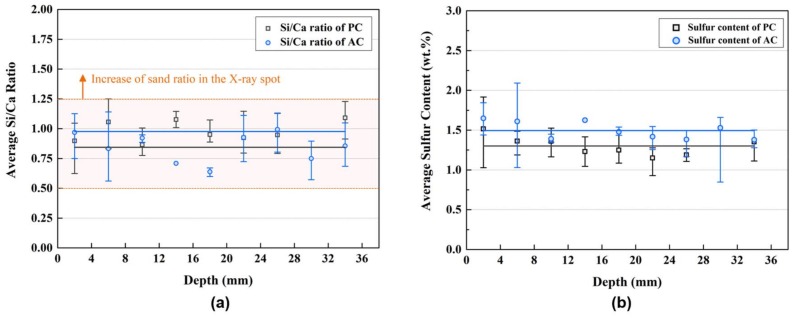
Chemical content of the mortar section: (**a**) Si/Ca ratio of the mortar; and (**b**) sulfur content of the mortar.

Figure 8 shows the Si/Ca ratio and sulfur content of the specimens according to the depth from the exposed surface. The Si/Ca ratio was considered to filter the measurement performed on the sand. The Si/Ca ratio of the C-S-H in the Portland cement paste is normally between 0.5 and 0.65 [33]. However, the increase in the Si/Ca ratio of the cement mortar is inevitable owing to the sand, which is mostly composed of SiO_2_. Additionally, the sulfur content of the matrix was considered for the reliability of the EDXRF results because the initial sulfur content of the matrix is not influenced by the chloride ingress [29]. 

Figure 6a shows the chloride profile of PC, which exhibits a normal trend of chloride profile in cement-based composites [6]. However, the chloride profile of AC appears close to the linear curve. The chloride ion content of AC was higher in the range of 8–28 mm from the surface; however, it was lower in the range of 0–8 mm and 28–34 mm than that of PC in the fitted curve, as shown in Figure 6b.

Although the chloride ion content of PC and AC did not exhibit a large difference, the chloride ion content of AR was higher than those of PC and AC at the same depth. Moreover, the chloride content of AR was much higher than that of the cement matrix (PC and AC) as it became closer to the exposed surface. The chloride ion content of the AR could be high because the AER has a higher driving force than that of the cement matrix, thus reducing the time to reach the adsorption equilibrium and increasing the chloride binding capacity [5,12,22,23]. The chloride adsorption capacity of the AER became higher with the increased chloride concentration in the solution test. Therefore, the chloride adsorption of the AER in mortar specimens could also be higher as it became closer to the exposed surface with high concentration of chloride.

The chloride ion contents of PC and AC were similar. However, the chloride ion content of AR was higher than that of the cement matrix (PC and AC). This indicates that the AER could adsorb the chloride ion from the pore solution and that it had a better performance in the chloride adsorption than in the hydration products in the matrix. However, it should be noted that the total chloride content that penetrated into the specimen could be higher in AC than in PC. Since the mortar specimens were immersed in the chloride solution for 140 days, the pore solutions were fully saturated. Chloride ions can be easily diffused owing to the driving force caused by concentration gradients [34]. In the case of AC, the diffused chloride ions were adsorbed more rapidly by the AER. This possibly increased the concentration gradients, thus resulting in higher chloride ingress in AC.

### 3.3. Electron Probe Microanalysis

**Figure 9:**
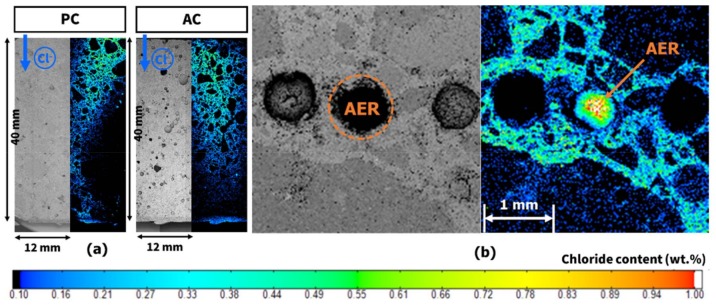
EPMA image: (**a**) chloride profile of the mortar sections; and (**b**) chloride content around the AER.

As the EDXRF results were obtained from manually selected spots, which makes their representativeness questionable, an image analysis of the entire area was performed using EPMA to supplement the EDXRF results. Figure 9a shows the chloride profile of the PC and AC sections measured by EPMA. Like in the EDXRF results, there was not a significant difference in the chloride ion content between PC and AC. Figure 9b shows the chloride ion content around the AER in the section of AC. The EPMA images clearly show the aspect of chloride ion ingress. The chloride ion content was higher in the AER than in the matrix around the AER. It confirms that the AER could adsorb the chloride ions passing through the pore. Furthermore, Figure 9b shows the direction of the concentration gradient of chloride ions toward the center of the AER. It was expected that the chloride adsorption of the AER from the pore solution was stronger than that of the hydration products in the matrix from pore solutions.

## 4. Conclusions

**Figure 10:**
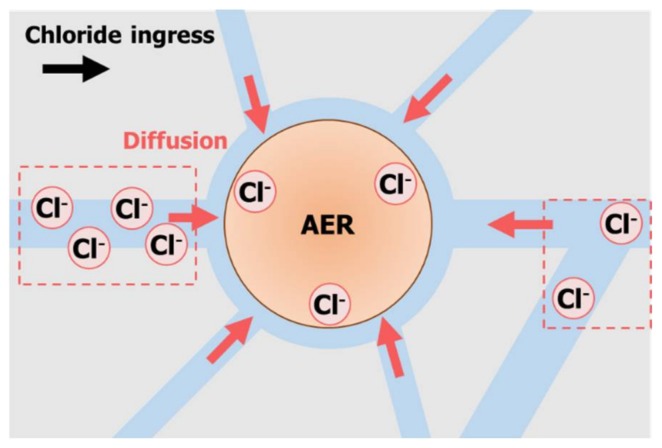
Schematic illustration of the chloride adsorption of the AER in the mortar specimen.

This study has demonstrated the kinetic and equilibrium behaviors of the AER in distilled water and a Ca(OH)_2_ saturated solution—both containing chloride ions. The effects of the AER on the chloride adsorption in the mortar specimen were investigated as well. The adsorption of chloride ion around the AER was dependent on the driving force induced by an electrostatic force of the AER and a concentration gradient of chloride ion. Figure 10 is a schematic illustration of the chloride adsorption behavior of the AER in the mortar specimen. The chloride ions around the AER diffused to the center of the AER regardless of the direction of chloride penetration. This suggests that the natural diffusion of chloride ions was interrupted, which delayed the chloride penetration through the pores. The following is a summary of the results:

1. The AER could adsorb chloride ions in both the distilled water and the Ca(OH)_2_ saturated solution. Since the hydroxyl ions could act as competing ions in the Ca(OH)_2_ saturated solution, which represent alkaline environments, the equilibrium time for adsorbing chloride ions was delayed and the adsorbed chloride ion content was reduced.
2. The chloride ion content was higher in the AER than in the matrix around the AER. However, the chloride ion content of the matrix was not largely different in the mortar specimens with and without the AER. The chloride ion content in the AER was higher than that in the matrix because the AER had a higher driving force than that of the cement matrix, thus reducing the time to reach the adsorption equilibrium and increasing the chloride binding capacity. The AER could successfully reduce the free chloride ions in the pore solution.

## Figures and Tables

**Table 1:** Properties of the anion exchange resin.

Item	Details
Appearance	Spherical bead
Matrix	Polystyrene divinylbenzene copolymer
Functional group	N^+^(CH_3_)_2_C_2_H_4_OH
Ionic form	OH^−^
Operating pH range	1–14
Specific gravity	1.13
Particle size range	300–1000 μm
Total capacity	1.3 eq·L^−1^

**Table 2:** Experimental conditions of the kinetic test in chloride solutions containing the AER.

Initial Cl^−^ Concentration (mg/L)	Water-to-AER Ratio (*w*/*w*)	Reaction Time (min)	Type of Solution
2000	40	1, 3, 10, 30, 60, and 120	Distilled water or Ca(OH)_2_ saturated solution

**Table 3:** Experimental conditions of the equilibrium test in chloride solutions containing the AER.

Reaction Time (min)	Water-to-AER Ratio (*w*/*w*)	Initial Cl^−^ Concentration (mg/L)	Type of Solution
120	40	100, 250, 500, 1000, 2000, 5000, and 15,000	Distilled water or Ca(OH)_2_ saturated solution

**Table 4:** Oxide composition of type 1 Portland cement.

Oxide Composition (wt %)	SiO_2_	Al_2_O_3_	Fe_2_O_3_	CaO	MgO	SO_3_	Loss of Ignition
Portland cement	19.29	5.16	2.87	61.68	4.17	2.53	2.49

**Table 5:** Mix proportions of cement mortar.

Type	Water-to-Cement Ratio	Sand-to-Cement Ratio	Content (wt %)	
			Sand	AER
PC	0.5	3	100	0
AC			98.32	1.68

**Table 6:** Experimental results of the kinetic test.

Type of Solution	Reaction Time (min)	Amount of Adsorbate (mg Cl^−^/g resin)				
		# of Test			Average	Coefficient of Variance
		1	2	3		
Distilled water	1	19.80	18.13	19.21	19.05	0.04
	3	19.08	23.86	23.91	22.28	0.12
	10	27.88	28.35	31.02	29.09	0.06
	30	27.04	29.30	31.21	29.18	0.07
	60	27.36	29.00	30.51	28.96	0.05
	120	29.00	27.65	27.36	28.00	0.03
Ca(OH)_2_ saturated solution	1	6.72	6.80	6.22	6.58	0.05
	3	9.12	4.85	11.04	8.34	0.38
	10	14.16	18.66	20.47	17.76	0.18
	30	20.44	23.75	23.86	22.68	0.09
	60	20.32	25.17	24.85	23.45	0.12
	120	25.89	25.35	25.30	25.51	0.01

**Table 7:** Experimental parameters of the pseudo first-order and pseudo second-order reaction equations.

Type of Solution	Parameters of the Pseudo First-Order Reaction Equation			Parameters of the Pseudo Second-Order Reaction Equation		
	*K*_1_ (min^−^^1^)	*Q_e_* (mg Cl^−^/g resin)	*R* ^2^	*K*_2_ (g resin/mg Cl^−^ min)	*Q_e_* (mg Cl^−^/g resin)	*R* ^2^
Distilled water	1.118	27.830	0.858	0.0676	28.619	0.950
Ca(OH)_2_ saturated solution	0.299	25.083	0.989	0.0129	25.965	0.997

**Table 8:** Experimental results of the equilibrium test.

Type of Solution	Initial Cl^−^ Concentration (mg/L)	Amount of Adsorbate (mg Cl^−^/g resin)				
		# of Test			Average	Coefficient of Variance
		1	2	3		
Distilled water	100	2.20	3.33	3.30	2.94	0.219
	250	6.80	7.22	8.01	7.34	0.084
	500	12.52	15.47	15.58	14.52	0.119
	1000	23.68	23.86	23.65	23.73	0.005
	2000	27.36	29.00	27.65	28.00	0.031
	5000	35.20	33.58	34.25	34.34	0.024
	15,000	50.27	57.77	42.29	50.11	0.155
Ca(OH)_2_ saturated solution	100	3.62	3.53	3.22	3.46	0.061
	250	7.29	5.46	7.30	6.68	0.159
	500	9.12	13.68	14.00	12.27	0.223
	1000	21.43	21.69	21.88	21.67	0.010
	2000	25.89	25.35	25.30	25.51	0.013
	5000	31.52	31.56	32.01	31.69	0.009
	15,000	38.53	49.20	52.80	46.85	0.158

**Table 9:** Experimental parameters of the Langmuir and Freundlich adsorption isotherm equations.

Type of Solution	Parameters of the Langmuir Adsorption Isotherm Equation			Parameters of the Freundlich Adsorption Isotherm Equation		
	*K_L_* (L/mg)	*Q_max_* (mg Cl^−^/g resin)	*R* ^2^	*K_F_* ((mg/g resin)/(mg/L)*^n^*)	*n*	*R* ^2^
Distilled water	0.00455	36.409	0.992	1.341	3.399	0.763
Ca(OH)_2_ saturated solution	0.00477	31.797	0.980	0.861	3.265	0.909
